# Impact of Seasonal and Organ-Related Fluctuations on the Anthelmintic Properties and Chemical Profile of *Cladium mariscus* (L.) Pohl Extracts

**DOI:** 10.3389/fpls.2022.934644

**Published:** 2022-06-23

**Authors:** Marta Oliveira, Caroline Sprengel Lima, Eulogio J. Llorent-Martínez, Hervé Hoste, Luísa Custódio

**Affiliations:** ^1^Centre of Marine Sciences, University of Algarve, Faro, Portugal; ^2^Laboratory of Antibiotics and Chemotherapeutics, Instituto de Biociências, Letras e Ciências Exatas (IBILCE), São Paulo State University, São José do Rio Preto, Brazil; ^3^Department of Physical and Analytical Chemistry, Faculty of Experimental Sciences, University of Jaén, Jaén, Spain; ^4^IHAP, Université de Toulouse, INRAE, ENVT, Toulouse, France; ^5^Université de Toulouse, ENVT, Toulouse, France

**Keywords:** halophytes, salt tolerant plants, anthelmintic, polyphenols, gastrointestinal nematodes, small ruminants

## Abstract

The use of plants and their metabolites stands as a promising option to tackle parasitic infections by gastrointestinal nematodes (GIN) in integrated control strategies. Still, the influence of environmental and phenological factors, and their interactions, in the wild on the metabolomics and biological properties of target plant species, is often disregarded. In this work, we hypothesized that variations in the anthelmintic (AH) properties and chemical composition of extracts from the salt tolerant species *Cladium mariscus* L. Pohl (sawgrass) may be influenced by seasonal factors and organ-parts. To test this hypothesis, acetone/water extracts were prepared from dried biomass obtained from aerial organs collected from sawgrass in consecutive seasons and tested against *Haemonchus contortus* and *Trichostrongylus colubriformis* by the larval exsheathment inhibition assay (LEIA) and egg hatching inhibition assay (EHIA). To ascertain the role of plant organ, the activity of leaves and inflorescences extracts from summer samples was compared. The role of polyphenols in the anthelmintic activity depending on GINs and fluctuations across seasons and plant organs was assessed using polyvinylpolypyrrolidone (PVPP), coupled with an in-depth chemical profiling analysis using high-performance liquid chromatography completed with electrospray ionization mass spectrometric detection (HPLC-ESI-MSn). Main differences in anthelmintic activities were observed for summer and autumn samples, for both assays. Moreover, inflorescences’ extracts were significantly more active than those from leaves against both parasite species on EHIA and against *H. contortus* on LEIA. Application of PVPP totally inhibit the AH effects based on EHIA and only partly for LEIA. Non-treated PVPP extracts were predominantly composed of flavan-3-ols, proanthocyanidins, luteolin and glycosylated flavonoids, while two flavonoid glycosides were quantified in all PVPP-treated samples. Thus, the activity of such compounds should be further explored, although some unknown metabolites remain to be identified. This study reinforces the hypothesis of the AH potential of sawgrass and of its polyphenolic metabolites uses as nutraceutical and/or phytotherapeutic drugs.

## Introduction

Parasitic infections by gastrointestinal nematodes (GIN) represent a serious economical and health threat to outdoor production systems of small ruminant worldwide, where *Haemonchus contortus*, *Teladorsagia circumcincta*, *Trichostrongylus* spp., and *Nematodirus* spp. are the most prevalent species (Charlier et al., 2018). As increasing anthelmintic resistances are reported, mainly due to the indiscriminate administration of available commercial synthetic drugs (Rose Vineer et al., 2020), it becomes peremptory to find novel GIN integrated control options. A promising suggested approach is the use of plants and their bioactive products, either as nutraceuticals, phytotherapeutic remedies, including essential oils as sources of secondary metabolites of veterinary interest to control parasites (Hoste et al., 2015).

Increasing evidence of *in vitro* and *in vivo* anthelmintic effects has been described for a range of botanical species and linked to a variety of bioactive metabolites, with particular emphasis on plants rich in polyphenolic compounds, specifically tannins (Hoste et al., 2006, 2015; Manolaraki, 2011; Spiegler et al., 2017; Santos et al., 2019; Liu et al., 2020). Such plants include both glycophytes, for example Legume forages (e.g., sainfoin: *Onobrychis viciifolia* Scop. and sulla: *Hedysarum coronarium* L.) and halophytes [e.g., mastic tree: *Pistacia lentiscus* L. and sawgrass: *Cladium mariscus* (L.) Pohl] (Oliveira et al., 2021c). Halophytes are high salt tolerant plants able to cope with several abiotic stressors, besides salinity, for example, high light intensity, high UV radiation, and drought/flood. That capacity is possible due several adaptation mechanisms, including the synthesis of high levels of antioxidant secondary molecules, such as polyphenols. Polyphenols are therefore produced as part of the plant defense machinery, and as such, the extent of its production is influenced by multiple complex abiotic and biotic interactions. Several studies on different models of bioactive plants rich in polyphenols have shown that the concentration of these compounds and, consequently, their pharmacological properties, rely to a range of factors either botanical (e.g., plant species, organs; plant cultivars and varieties, phenological stages), geographical and environmental (e.g., season, area of collection), as well as cultivation systems (Manolaraki, 2011). Similar factors have been described for halophytes wild plants. For example, high light intensity, UV irradiance, and saline stress enhanced the biosynthesis of antioxidant flavonoids in *Ligustrum vulgare* L., particularly quercetin and luteolin glycosides (Tattini et al., 2004; Agati et al., 2011). Variations on the chemical composition and bioactive properties of plant organs (e.g., leaves, stems, flowers) are described for different salt-tolerant species such as *Eryngium maritimum* L., *Limoniastrum monopetalum* L., and *Limonium algarvense* L. (Trabelsi et al., 2012; Rodrigues et al., 2015; Pereira et al., 2019). In addition, Azaizeh et al. (2013, 2015) observed that the polyphenol content and anthelmintic effects of *P*. *lentiscus* L. and *Inula viscosa* L. extracts on third stage (L3) mixed-species larvae exsheathment process changes across seasons. Thus, seasonality and organ-related variations should be taken into consideration when evaluating the anthelmintic value of botanical species aiming at its future use for GIN control strategies.

**FIGURE 1:**
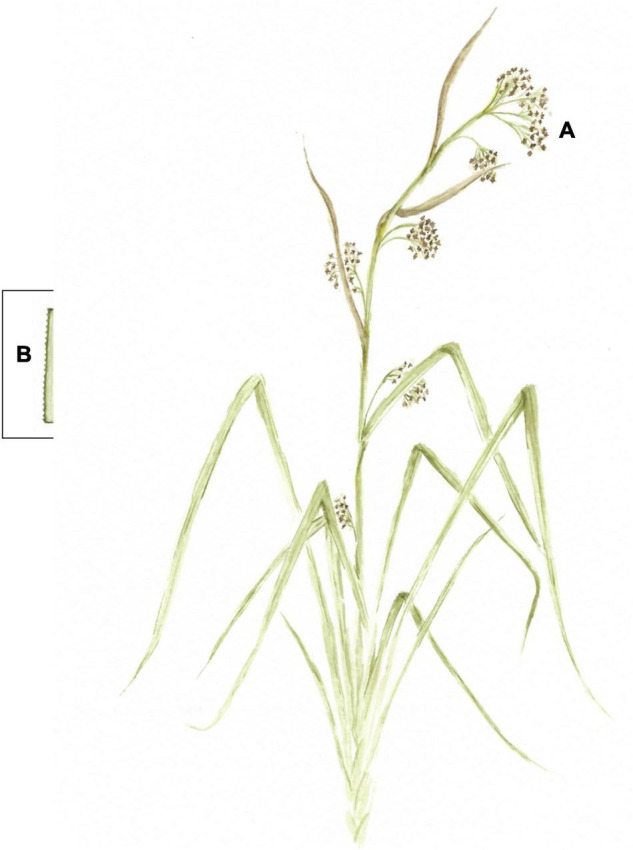
Illustration of *Cladium mariscus* (L.) Pohl (sawgrass) aerial organs: inflorescences **(A)** and detail of leaves **(B)**. Illustration made by M. Oliveira.

*Cladium mariscus* (L.) Pohl (Cyperaceae), also known as sawgrass, is a grass-like perennial halophytic herbaceous species distributed along the Mediterranean region, in low to moderate saline environments (Gerdol et al., 2018). Sawgrass lengthy and long-lasting leaves have characteristic saw-shaped margins and inflorescences rise above leaves (Castroviejo, 2008; Figure 1). In our previous investigations, sawgrass 80% acetone/water extracts exhibited high polyphenol content coupled with *in vitro* antioxidant and anti-inflammatory properties, with marked differences among seasons (Lopes et al., 2016; Oliveira et al., 2021a). Moreover, sawgrass aerial organs extract suppressed egg hatching and L3 larvae exsheathment processes of two models of GINs of sheep and goats, namely one abomasal species *Haemonchus contortus* and one intestinal species *Trichostrongylus colubriformis* (Oliveira et al., 2021b).

In this work, we hypothesize that (1) differences in anthelmintic effects can occur among seasons and between aerial organs of sawgrass, and that (2) polyphenols could have a role on the detected anthelmintic effects. The first hypothesis was explored by using sawgrass extracts prepared from biomass collected from wild plants during the four seasons, and from different plant organs, namely leaves and inflorescences. Extracts were *in vitro* tested relying on two nematode stages (egg hatching and L3 larvae), and two key nematode species (*H. contortus* and *T. colubriformis*). To test the second hypothesis, polyvinylpolypyrrolidone (PVPP), a polyphenol-binding agent, was added to the extracts and the metabolites in non-treated and treated samples were profiled by high-performance liquid chromatography with electrospray ionization mass spectrometric detection (HPLC-ESI-MSn).

## Materials and Methods

### Plant Collection and Processing

*Cladium mariscus* (L.) Pohl aerial parts, including leaves and inflorescences (Figure 1), were manually harvested in Ludo, Faro, southern Portugal (37°01’03.3” N, 7°59’18.1”W, aprox. 7 m elevation) in spring (Sp; April, 2017: minimum temperature: 12°C, maximum temperature: 21°C, mean precipitation: 34 mm), summer (Su; July 2017: minimum temperature: 19°C, maximum temperature: 29°C; mean precipitation: 1 mm), autumn (Au; October, 2017: minimum temperature: 14°C, maximum temperature: 25°C, mean precipitation: 55 mm) and winter (Wi; January, 2018: minimum temperature: 8°C, maximum temperature: 16°C; mean precipitation: 210 mm) (source: WeatherSpark based on data obtained in the Faro International Airport). Inflorescences were present during summer and, to a lesser extent, in autumn, while green leaves were collected all year. In addition, only for summer season, samples of leaves (Le) and inflorescences (Inf) were also collected, for separate analysis. Afterward, samples were transported to the laboratory, washed, frozen at -20°C until freeze-dried (Lyoalfa 15) for 3 days and reduced to powder using a coffee and a ball miller (Retsch PM 100). For plant collection in the wild, mandatory licenses for the Portuguese territory were obtained, and the protocol for collection was followed according to the standard procedures recommended by “Instituto da Conservacão da Natureza e das Florestas (ICNF),” the national regulatory body. Voucher specimen (XBH03) were kept in the XtremeBio group herbarium, at Centre of Marine Sciences (CCMAR), University of Algarve (UAlg), Faro, Portugal, and the formal identification of the botanical material was made by Dr. Luiìsa Custoìdio (CCMAR).

### Sample Preparation

Dried biomass was extracted with an 80% aqueous acetone solution (1:40, *w/v*) at room temperature (RT, 20–25°C), for 16 h, under stirring. Then, the residue was sieved using qualitative filters and concentrated in a rotary evaporator under reduced pressure and temperature (approximately 40°C), aiming acetone removal. Afterward, the aqueous residue was freeze-dried, and the extracts recovered for use in the further anthelmintic and chemical assays.

### *In vitro* Anthelmintic Assays

#### *Haemonchus contortus* and *Trichostrongylus colubriformis* Parasites

The feces of caprine and ovine donors, kept indoors, monospecifically infected with susceptible strains of either *H. contortus* or *T. colubriformis*, were used to obtain third stage larvae (L3) and eggs. The facilities hosting the animals and trial performance met French ethical and welfare rules (agreement C 31 555 27 of August 19, 2010). Larvae were recovered with the Baermann method, stored in culture flasks at 4°C for one (*H. contortus*) or 4 months (*T. colubriformis*), before use in the larval exsheathment inhibition (LEIA) experiments. Eggs were collected on the day of the egg hatching inhibition (EHIA) experiments and used up to 2 h prior to collection.

#### Larval Exsheathment Inhibition Assay

Larval exsheathment inhibition assay (LEIA) protocol was performed as previously described by Bahuaud et al. (2006). The extracts were prepared in serial concentrations of 1,200, 600, 300, 150, and 75 μg/mL in phosphate buffered saline (PBS; 0.1 M phosphate, 0.05 M NaCl, pH 7.2) and incubated at 23°C for 3 h with L3 larvae (approx. 800 larvae/mL). After three times washed and centrifuged, the pellet was resuspended in 200 μL of PBS, and 40 μL were used to count the proportion of ensheathed/exsheathed larvae at 0 min. Then, the remaining 160 μL were exposed to a solution of Milton (2% *w/v* sodium hypochlorite, 16.5% *w/v* sodium chloride in PBS), aiming at the artificial induction of larvae exsheathment. The optimal concentration of Milton’s solution to achieve a gradual exsheathment process, i.e., reaching 100% exsheathment in 60 min, was tested for each batch before use. After 20, 40, and 60 min of exposure, the number of ensheathed and exsheathed larvae were counted under a microscope (400×). For each extract concentration and negative control (PBS) four replicates were performed and run in parallel. Percentage of larvae exsheathment (LE) for each replicate, was given by the formula: %LE = [(number of exsheathed larvae)/(number of exsheathed + ensheathed larvae) × 100].

#### Egg Hatching Inhibition Assay

Egg hatching inhibition assay (EHIA) protocol was performed as previously described in Oliveira et al. (2021b). Fecal samples were filtrated twice using a gaze hydrophile compress, sieved (25 μm) and washed with distilled water. The residues were subjected to three centrifugation steps using a saline saturated solution (*d* = 1.2), and the pellet recovered in PBS. After quantification, eggs (100 per well) were plated in 48-well sterile plates and treated with the extract solutions, prepared in PBS in concentrations ranging from 5,000 to 78 μg mL^–1^. After a 48 h incubation period at 27°C, the number of larvae and eggs, in each well, was registered after microscopic counting. Six replicates were performed for each extract concentration and the negative control, PBS, was run in parallel. The percentage of egg hatching (EH) for each well, was obtained as given by the following formula: %EH = [(number of larvae)/(number of eggs + larvae) × 100].

### Polyvinylpolypyrrolidone Treatment

To appraise the possible role of polyphenols in the anthelmintic activity, PVPP, a polyphenol inhibitor (Doner et al., 1993) was added to extracts solutions (ratio 50:1; *w/w*) at the concentrations of 2,500 μg mL^–1^ and 1,200 μg mL^–1^, as previously described by Oliveira et al. (2021b). After overnight incubation at 4°C, the solution was centrifuged for 10 min at 4,500 rpm and the supernatant posteriorly used in the *in vitro* assays and for chemical analysis. The extracts not exposed to PVPP and the negative control, PBS, were run in parallel.

### Chemical Profiling by High-Performance Liquid Chromatography With Electrospray Ionization Mass Spectrometric Detection

Chromatographic analyses were performed with an Agilent Series 1100 HPLC system with a G1315B diode array detector (Agilent Technologies, Santa Clara, CA, United States) and an ion trap mass spectrometer (Esquire 6000, Bruker Daltonics, Billerica, MA, United States) with an electrospray interface. Separation was performed in a Luna Omega Polar C_18_ analytical column (150 × 3.0 mm; 5 μm particle size) with a Polar C_18_ Security Guard cartridge (4 × 3.0 mm), both purchased from Phenomenex, Torrance, CA, United States. Detailed chromatographic conditions are available in Fernández-Poyatos et al. (2019). Compounds’ identification was performed by mass spectrometry data, whereas the quantification was carried out by UV using analytical standards of catechin (280 nm), apigenin (350 nm), kaempferol (350 nm) and luteolin (350 nm). Calibration graphs were constructed in the 0.5–100 mg/mL range. Repeatability (*n* = 9) and intermediate precision (*n* = 9, 3 consecutive days) were lower than 3 and 8%, respectively. Each analytical standard was used to quantify the corresponding compound or compounds of the same chemical family. The characterization of the metabolites was carried out by HPLC-ESI-MSn using the negative ion mode and identification was performed using analytical standards as well as bibliographic information. Compounds were numbered according to their elution order, keeping the same numbering in all extracts.

### Statistical Analysis

Results are expressed either as effective concentration that inhibits 50% of the larval exsheathment and/or egg hatching (EC_50_) values (μg/mL) including 95% confidence intervals (CI). EC_50_ values and 95% CI were obtained by Probit analysis, while significant differences among groups were detected by relative median potency estimates, through IBM SPSS Statistics v. 26.0 software. PVPP results are expressed as average ± standard error of the mean (SEM).

## Results

### Season- and Organ-Related Effects on *in vitro* Anthelmintic Properties

Table 1 summarizes the *in vitro* anthelmintic activity results of sawgrass extracts prepared from biomass collected in successive seasons, against *H. contortus* and *T. colubriformis* larvae and eggs. Concerning the LEIA results, no significant seasonal effects were recorded for *H. contortus*, however, the summer sample was the most effective on inhibiting *T. colubriformis* L3 larvae exsheathment (EC_50_ = 77.8 μg mL^–1^), followed by the autumn extract (IC_50_ = 110.9 μg mL^–1^). Spring and winter samples were less active on *T. colubriformis* than *H. contortus*, while summer and autumn samples inhibited larvae exsheathment from both species in a similar manner. The extracts consistently presented lower EC_50_ values toward L3 larvae exsheathment than egg hatching processes and were more effective on impairing egg hatching of *H. contortus* than those of *T. colubriformis* (Table 1). While no seasonal effects were observed on EHIA using *T. colubriformis* parasites, the summer extract was the most active against *H. contortus* (EC_50_ = 1446.6 μg mL^–1^). The inflorescence extract was significantly more active to inhibit larvae exsheathment of *H. contortus* (EC_50_ = 60.0 μg mL^–1^) than leaves (EC_50_ = 87.7 μg mL^–1^). Indeed, the inflorescences extracts were also more effective than leaves against egg hatching, as illustrated by EC_50_ values for inflorescence representing 37% of those obtained for leaves, for both parasite species (Table 2). Interestingly, despite the high activity of inflorescences sample on EHIA, this is not reflected in the seasonal samples, particularly in summer as marked differences are not observed between samples collected in different seasons.

**TABLE 1:** Effective concentration that inhibits 50% of larval exsheathment or egg hatching (EC_50_ values, μg mL^–1^) and 95% confidence intervals (CI) obtained for *Cladium mariscus* (L.) Pohl (sawgrass) extracts on *Haemonchus contortus* and *Trichostrongylus colubriformis* L3 larvae exsheathment (LEIA) and egg hatching inhibition assays (EHIA).

	LEIA		EHIA	
	*H. contortus*	*T. colubriformis*	*H. contortus*	*T. colubriformis*
Spring	94.0*^Aa^* (72.3–123.4)	159.6*^Bb^* (125.7–211.3)	1902.8*^Ab^* (1712.9–2126.3)	2275.3*^Ba^* (2028.9–2564.6)
Summer	88.9*^Aa^* (66.3–118.7)^†^	77.8*^Aa^* (60.6–100.0)^†^	1496.6*^Aa^* (1326.5–1698.9)^†^	2575.5*^Ba^* (2324.1–2881.8)^†^
Autumn	99.6*^Aa^* (77.8–128.3)	110.9*^Aab^* (88.4–140.8)	1826.3*^Ab^* (1622.9–2069.8)	2384.6*^Ba^* (2118.3–2704.7)
Winter	70.4*^Aa^* (52.2–97.0)	128.9*^Bb^* (97.2–176.3)	1873.2*^Ab^* (1688.5–2087.1)	2386.3*^Ba^* (2137.5–2681.7)

*^†^Oliveira et al. (2021b). For each assay, different letters represent significant statistical differences (P < 0.05) either between GIN species (capital letter within rows) and/or between seasons (small letters; within columns), respectively, based on relative median potency estimates.*

**TABLE 2:** Effective concentration that inhibits 50% of larval exsheathment or egg hatching (EC_50_ values, μg mL^–1^) and 95% confidence intervals (CI) obtained for *Cladium mariscus* (L.) Pohl (sawgrass) organ extracts on *Haemonchus contortus* and *Trichostrongylus colubriformis* L3 larvae exsheathment (LEIA) and egg hatching inhibition assays (EHIA).

	LEIA		EHIA	
	*H. contortus*	*T. colubriformis*	*H. contortus*	*T. colubriformis*
Leaves	87.7*^Ab^* (71.3–108.5)	81.1*^Aa^* (67.2–98.5)	2079.4*^Ab^* (1927.8–2248.1)	2289.9*^Ab^* (2118.7–2481.3)
Inflorescences	60.0*^Aa^* (47.6–74.9)	78.6*^Aa^* (64.6–96.2)	776.5*^Aa^* (732.3–824.7)	848.2*^Aa^* (797.6–903.5)

*Different letters represent significant statistical differences among GIN species (capital; rows) and plant organs (small; columns) for each assay, respectively, based on Relative median potency estimates.*

### Role of Polyphenols in the Anthelmintic Activity: Polyvinylpolypyrrolidone as a Polyphenol Binding Agent

**FIGURE 2:**
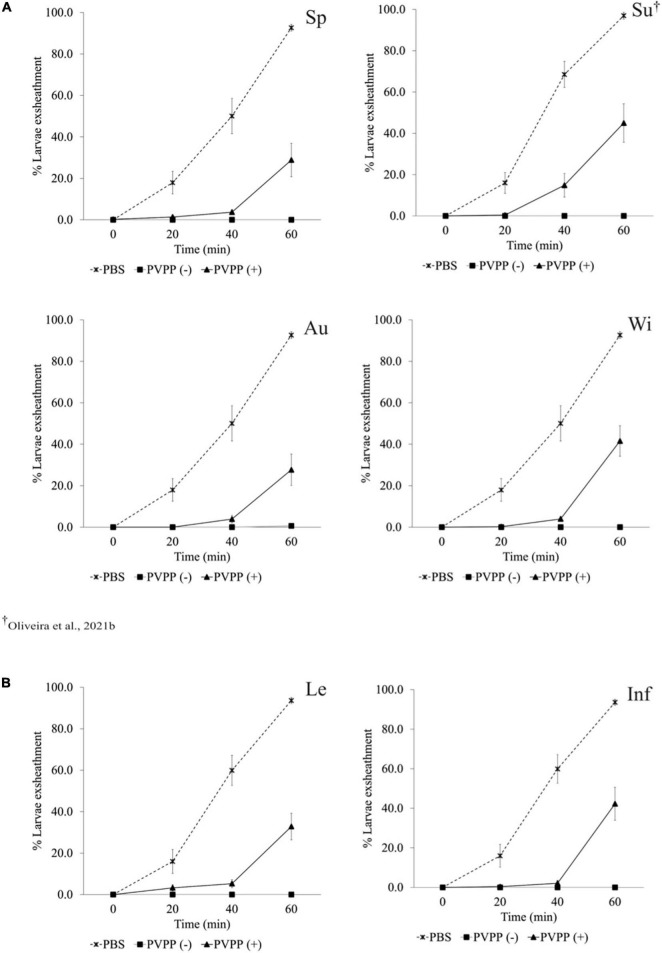
Anthelmintic effects of *Cladium mariscus* (L.) Pohl (sawgrass) seasonal **(A)** and organ **(B)** extracts, on LEIA for *Haemonchus contortus* at the concentration of 1,200 μg mL^–1^, either treated [PVPP(+)] or not [PVPP(-)] with PVPP. Sp, spring; Su, summer; Au, autumn; Wi, winter; Le, leaves; Inf, inflorescences.

**FIGURE 3:**
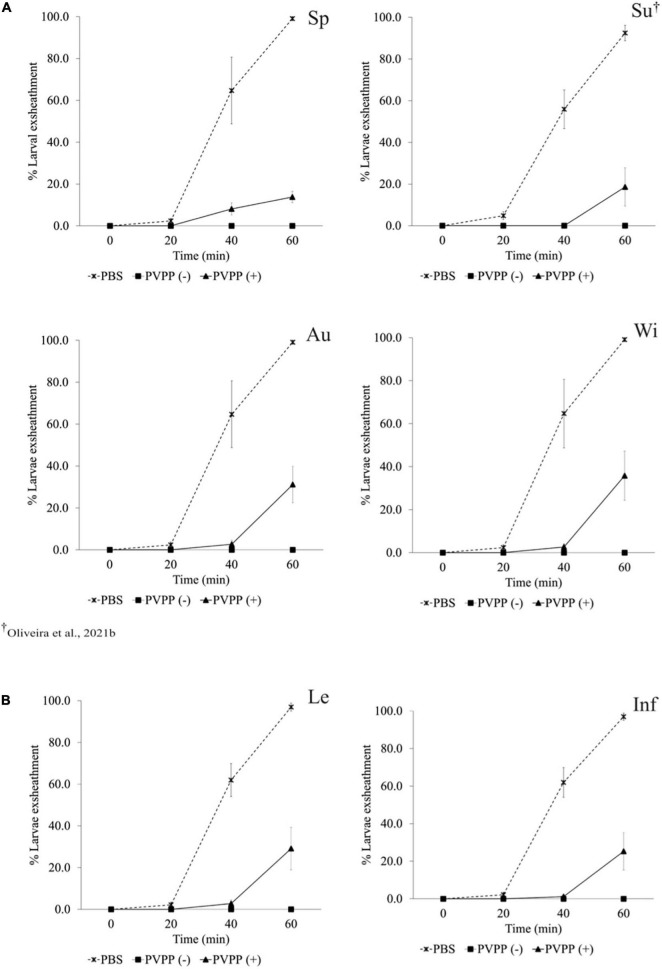
Anthelmintic effects of *Cladium mariscus* (L.) Pohl (sawgrass) seasonal **(A)** and organ **(B)** extracts, on LEIA for *Trichostrongylus colubriformis* at the concentration of 1,200 μg mL^–1^, either treated [PVPP(+)] or not [PVPP(-)] with PVPP. Sp, spring; Su, summer; Au, autumn; Wi, winter; Le, leaves; Inf, inflorescences.

**FIGURE 4:**
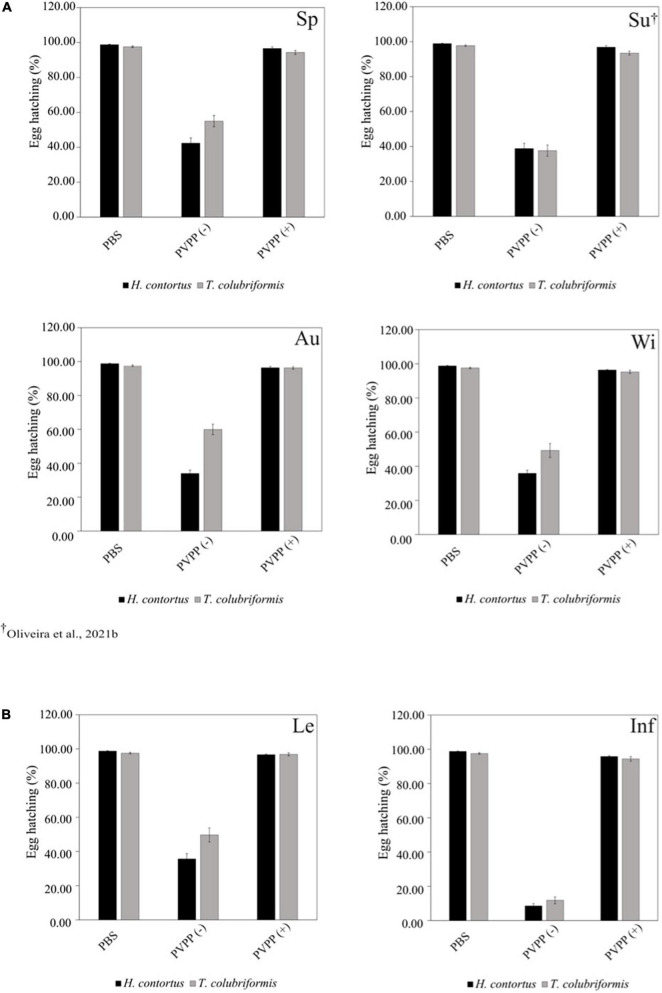
Anthelmintic effects of *Cladium mariscus* (L.) Pohl (sawgrass) seasonal **(A)** and organ **(B)** extracts on EHIA for *Haemonchus contortus* and *Trichostrongylus colubriformis* at the concentration of 2,500 μg mL^–1^, either treated [PVPP(+)] or not [PVPP(-)] with PVPP. Sp, spring; Su, summer; Au, autumn; Wi, winter; Le, leaves; Inf, inflorescences.

All PVPP-treated extracts, including those from leaves and inflorescences, still exhibit activity against the *H. contortus* and *T. colubriformis* L3 exsheathment in a similar manner (10–40% larvae exsheathment), while egg hatching was completely restored (Figures 2–4).

### High-Performance Liquid Chromatography With Electrospray Ionization Mass Spectrometric Detection Comparative Analysis of the Chemical Profile of Polyvinylpolypyrrolidone Treated vs. Non-treated Extracts

The chemical characterization of the metabolites in each extract carried out by HPLC-ESI-MSn analysis is depicted in Table 3, while quantification of the main compounds of non-treated and treated PVPP samples is presented in Tables 4, 5. Compounds were numbered according to their elution order, keeping the same numbering in all extracts.

**TABLE 3:** Characterization of the polyphenolic compounds found in the analyzed extracts of *Cladium mariscus* (L.) Pohl (sawgrass).

No.	t*_*R*_* (min)	[M-H]^–^ *m/z*	m/z (% base peak)	Assigned identification	PVPP (-)	PVPP (+)
1	1.8	377	MS^2^ [377]: 341 (100) MS^3^ [377→341]: 179 (100), 161 (24), 143 (13), 119 (25), 113 (20)	Disaccharide (HCl adduct)	All samples	All samples
2	4.6	305	MS^2^ [305]: 261 (7), 221 (43), 219 (72), 179 (100), 165 (35)	(Epi)gallocatechin	Su, Au, Wi, Le, Inf	Absent
3	7.0	577	MS^2^ [577]: 451 (38), 425 (100), 407 (96), 305 (21), 289 (45), 287 (17)	Proanthocyanidin dimer	All samples	Absent
4	7.2	305	MS^2^ [305]: 261 (12), 221 (55), 219 (77), 179 (100), 165 (26)	(Epi)gallocatechin	All samples	Absent
5	8.8	289	MS^2^ [289]: 245 (100), 205 (43), 203 (28), 179 (24)	Catechin	All samples	Absent
6	9.0	353	MS^2^ [353]: 191 (100), 179 (3), 173 (4), 135 (1)	Chlorogenic acid	All samples	All samples
7	9.3	865	MS^2^ [865]: 739 (54), 713 (41), 695 (100), 577 (52), 451 (29), 407 (54), 405 (23), 289(19), 287 (41)	Proanthocyanidin trimer	Su, Wi, Le, Inf	Absent
8	9.5	429	MS^2^ [429]: 267 (100)	Unknown	All samples	All samples
9	9.9	577	MS^2^ [577]: 451 (69), 441 (17), 425 (30), 305 (100), 289 (10), 287 (8)	Proanthocyanidin dimer	All samples	Absent
10	10.1	865	MS^2^ [865]: 739 (76), 695 (100), 577 (83), 451 (18), 407 (97), 287 (58)	Proanthocyanidin trimer	Su, Au, Inf	Absent
11	10.1	561	MS^2^ [561]: 543(18), 435 (58), 409 (73), 425 (46), 289 (100), 271 (41) MS^3^ [561→289]: 245 (100), 205 (57), 203 (30)	Proanthocyanidin dimer	All samples	Absent
12	10.9	577	MS^2^ [577]: 451 (25), 441 (9), 425 (100), 407 (61), 305 (43), 289 (33), 287 (10)	Proanthocyanidin dimer	All samples	Absent
13	11.5	577	MS^2^ [577]: 451 (28), 425 (10), 305 (100), 289 (4), 287 (6)	Proanthocyanidin dimer	All samples	Absent
14	12.1	289	MS^2^ [289]: 245 (100), 205 (48), 203 (19), 179 (25), 161 (10)	Epicatechin	All samples	Absent
15	13.7	579	MS^2^ [579]: 561 (16), 519 (16), 489 (100), 459 (99), 429 (18), 399 (50), 369 (14)	Luteolin-*C*-hexoside-*C*-pentoside	All samples	All samples
16	15.9	563	MS^2^ [563]: 545 (14), 503 (15), 473 (48), 443 (100), 383 (37), 353 (43)	Apigenin-*C*-hexoside-*C*-pentoside	All samples	All samples
17	16.5	447	MS^2^ [447]: 429 (14), 357 (70), 327 (100), 285 (3)	Luteolin-6-*C*-glucoside (isoorientin)	All samples	Sp, Su
18	17.0	461	MS^2^ [461]: 341 (100), 313 (66), 298 (37)	Unknown	All samples	All samples
19	17.0	549	MS^2^ [549]: 531 (12), 489 (26), 459 (100), 441 (13), 429 (10), 399 (64), 369 (25)	Luteolin 6-*C*-pentosyl-8-*C*-pentoside	All samples	All samples
20	17.3	563	MS^2^ [563]: 503 (22), 473 (100), 443 (69), 383 (61), 353 (97)	Apigenin-*C*-hexoside-*C*-pentoside	Sp, Su, Au, Wi, Le	Sp, Su, Le, Inf
21	20.2	463	MS^2^ [463]: 317 (100) MS^3^ [463→317]: 271 (100), 151 (21)	Myricetin-*O*-deoxyhexoside	Sp, Au	Absent
22	20.2	431	MS^2^ [431]: 341 (44), 311 (100), 283 (5) MS^3^ [431→311]: 283 (100)	Apigenin-8-*C*-glu (vitexin)	Sp, Su, Au, Wi, Le	Absent
23	21.4	447	MS^2^ [447]: 285 (100) MS^3^ [447→285]: 285 (100), 241 (47), 151 (10)	Kaempferol-*O*-hexoside	All samples	Absent
24	22.2	417	MS^2^ [417]: 399 (22), 357 (100), 327 (49) MS^3^ [417→357]: 339 (100), 311 (24), 297 (82), 285 (93)	Luteolin-*C*-pentoside	Sp, Su, Au, Wi, Le	Absent
25	22.8	243	MS^2^ [243]: 225 (100), 201 (50), 199 (23), 157 (20)	Unknown	Su, Inf	Absent
26	24.9	485	MS^2^ [485]: 375 (100), 361 (27) MS^3^ [485→375]: 357 (87), 333 (70), 329 (100), 313 (64)	Unknown	Su, Au, Inf	Absent
27	26.9	317	MS^2^ [317]: 179 (100), 151 (47)	Myricetin	Sp, Su, Au, Wi, Le	Absent
28	32.1	485	MS^2^ [485]: 375 (100), 357 (13) MS^3^ [485→375]: 357 (100)	Unknown	Su, Au, Inf	Absent
29	36.0	285	MS^2^ [285]: 285 (100), 267 (5), 243 (2), 241 (3)	Luteolin	All samples	Absent

*Sp, spring; Su, summer; Au, autumn; Wi, winter; Le, leaves; Inf, inflorescences.*

**TABLE 4:** Quantification of the main compounds detected in *Cladium mariscus* (L.) Pohl (sawgrass) before PVPP sample treatment.

No.	Assigned identification	mg g^–1^ DE					
		Sp	Su	Au	Wi	Le	Inf
Catechin derivatives							
3 + 4	Proanthocyanidin dimer + (epi)gallocatechin	4.5 ± 0.3*^b^*	2.8 ± 0.2*^ab^*	30 ± 2*^d^*	9.2 ± 0.6*^c^*	2.1 ± 0.1*^a^*	0.84 ± 0.05*^a^*
14	Epicatechin	0.57 ± 0.04*^a^*	3.4 ± 0.2*^b^*	5.3 ± 0.4*^c^*	0.22 ± 0.02*^a^*	0.17 ± 0.01*^a^*	6.1 ± 0.4*^d^*
**Total**		**5.1 ± 0.3*^b^***	**6.2 ± 0.3*^b^***	**35 ± 2*^d^***	**9.4 ± 0.6*^c^***	**2.3 ± 0.1*^a^***	**6.9 ± 0.4*^b^***
Flavonoids							
15	Luteolin-*C*-Hex-*C*-Pen	2.9 ± 0.2*^b^*	2.8 ± 0.2*^b^*	10.1 ± 0.6*^c^*	3.4 ± 0.2*^b^*	3.6 ± 0.2*^b^*	0.55 ± 0.04*^a^*
16	Apigenin-*C*-Hex-*C*-Pen	0.43 ± 0.02*^a^*	0.43 ± 0.03*^a^*	1.5 ± 0.1*^c^*	0.70 ± 0.04*^b^*	0.43 ± 0.03*^a^*	0.32 ± 0.02*^a^*
17	Luteolin-6-*C*-glucoside (isoorientin)	2.3 ± 0.1*^bc^*	2.6 ± 0.1*^c^*	8.3 ± 0.4*^e^*	2.0 ± 0.1*^b^*	3.7 ± 0.2*^d^*	0.56 ± 0.03*^a^*
21 + 22	Myricetin-*O*-dHex + vitexin	0.53 ± 0.04*^b^*	0.41 ± 0.03*^ab^*	1.7 ± 0.1*^c^*	0.35 ± 0.3*^a^*	0.36 ± 0.03*^a^*	–
23	Kaempferol-*O*-Hex	0.87 ± 0.06*^b^*	1.19 ± 0.07*^c^*	3.8 ± 0.2*^d^*	1.2 ± 0.1*^c^*	1.36 ± 0.08*^c^*	0.43 ± 0.03*^a^*
24	Luteolin-*C*-Pen	0.63 ± 0.04*^a^*	1.02 ± 0.06*^c^*	1.8 ± 0.1*^d^*	0.62 ± 0.04*^a^*	0.82 ± 0.05*^b^*	–
27	Myricetin	0.27 ± 0.02*^a^*	0.31 ± 0.02*^ab^*	0.44 ± 0.03*^c^*	0.75 ± 0.05*^d^*	0.39 ± 0.03*^bc^*	–
29	Luteolin	0.31 ± 0.02*^a^*	4.1 ± 0.2*^d^*	3.5 ± 0.2*^c^*	0.86 ± 0.06*^b^*	0.78 ± 0.05*^ab^*	6.1 ± 0.3*^e^*

**Total**		**8.2 ± 0.2*^a^***	**12.9 ± 0.3*^d^***	**31.1 ± 0.8*^e^***	**9.9 ± 0.4*^b^***	**11.4 ± 0.3*^c^***	**8.0 ± 0.3*^a^***
**TIPC**		**13.3 ± 0.4*^a^***	**19.1 ± 0.4*^b^***	**66 ± 2*^c^***	**19.3 ± 0.7*^b^***	**13.7 ± 0.3*^a^***	**14.9 ± 0.5*^a^***

*DE, dry extract; Sp, spring; Su, summer; Au, autumn; Wi, winter; Le, leaves; Inf, inflorescences. Bold values represent the sum of each type of components. TIPC, total individual phenolic content (sum of all compounds quantified individually). –: not detected. Different superscript letters correspond to significant differences between seasons (p < 0.05).*

**TABLE 5:** Quantification of the main compounds detected in *Cladium mariscus* (L.) Pohl (sawgrass) after PVPP sample treatment.

No.	Assigned identification	mg g^–1^ DE					
		Sp	Su	Au	Wi	Le	Inf
Flavonoids							
15	Luteolin-*C*-Hex-*C*-Pen	0.67 ± 0.05*^cd^*	0.51 ± 0.03*^b^*	0.51 ± 0.04*^b^*	0.61 ± 0.04*^bc^*	0.77 ± 0.05*^d^*	0.26 ± 0.02*^a^*
16	Apigenin-*C*-Hex-*C*-Pen	0.31 ± 0.02*^ab^*	0.28 ± 0.02*^ab^*	0.30 ± 0.02*^ab^*	0.33 ± 0.02*^b^*	0.33 ± 0.02*^b^*	0.26 ± 0.02*^a^*

**TIPC**		**0.98 ± 0.05*^c^***	**0.79 ± 0.03*^b^***	**0.81 ± 0.04*^b^***	**0.94 ± 0.04*^c^***	**1.1 ± 0.05*^d^***	**0.52 ± 0.03*^a^***

*DE, dry extract; Sp, spring; Su, summer; Au, autumn; Wi, winter; Le, leaves; Inf, inflorescences. Bold values represent the sum of each type of components. TIPC, total individual phenolic content (sum of all compounds quantified individually). Different superscript letters correspond to significant differences between seasons and plant organs (p < 0.05).*

All extracts were mainly composed of flavonoids, specifically flavan-3-ols (epigallocatechin, epicatechin 14) and proanthocyanidins (condensed tannins; 2.3–35.3 mg g-1, DW), luteolin (0.31–6.1 mg g-1, DW) and glycosylated flavonoids (1.9–27.6 mg g-1 DW; Table 4). Summer and autumn samples had the highest amounts of epicatechin 14 (3.4–5.3 mg g -1, DW) and of luteolin 29 (3.5–4.1 mg g-1, DW). Compound **5** was identified as catechin by comparison with an analytical standard. Compound 14, with the same fragmentation pattern, was thus identified as epicatechin. Several compounds were characterized as (epi)catechin derivatives. Compounds **2** and **4** were characterized as (epi)gallocatechin isomers. Compounds **3**, **7**, and **9–13** were characterized as proanthocyanidin dimers and trimers (Kajdžanoska et al., 2010; Hamed et al., 2014).

Compounds **16** and **20**, with [M-H]- at m/z 563, were characterized as apigenin-*C*-hexoside-*C*-pentoside isomers due to the fragment ions observed at [M-H- 60]-, [M-H- 90]-, [M-H- 120]-, [M-H- 180]-, and [M-H- 210]-, characteristic of di-C-glycoside flavonoids (Han et al., 2008). Compound **22** was characterized as apigenin-8-*C*-glucoside (vitexin) based on bibliographic information (Waridel et al., 2001). Compound **23** was characterized as kaempferol-*O*-hexoside due to the neutral loss of 162 Da to yield kaempferol aglycone at m/z 285. The fragmentation of kaempferol was compared with an analytical standard. Compound **27** was identified as myricetin, with deprotonated molecular ion at m/z 317 and fragment ions at m/z 179 and 151. Compound **21** was characterized as myricetin-*O*-deoxyhexoside. Compound **29** was identified as luteolin by comparison with an analytical standard. Compounds **15**, **17**, **19**, and **24** were luteolin-*C*-glycosides. Compound **17** was characterized as isoorientin due to the fragment ion at m/z 429 (absent in orientin) (Algamdi et al., 2011). Besides flavonoids, one phenolic acid, compound **6** was identified as chlorogenic acid by comparison with an analytical standard. Moreover, compound **1** suffered the neutral loss of 36 Da (HCl) to yield the base peak at m/z 341. Its fragmentation pattern was consistent with a disaccharide formed by two hexosides (probably glucose) (Brudzynski and Miotto, 2011).

The inflorescences extract (from the summer sample) exhibited increased amounts of compounds **14** and **29** (6.1 and 6.1 mg g^–1^ DW), in contrast to leaves (0.17 and 0.78 mg g^–1^ DW). The proanthocyanidin dimer + (epi)gallocatechin **3** + **4** reached a maximum of 30 mg g^–1^, dry weight (DW) in autumn. High amounts of **3 + 4** are noted in all extracts ranging from 2.1 up to 30 mg g^–1^ DW, except for inflorescences (0.84 mg g^–1^ DW). Proanthocyanidins **7** and **9–13** were identified but not quantified due to low signal.

## Discussion

Salt-tolerant plants (halophytes) have ethnomedicinal and ethnoveterinary reported uses and are considered important sources of compounds and products with multiple commercial uses. Such plants may also represent an important resource for animal management and veterinary purposes, in view of the growing need to identify alternatives to chemical ingredients in livestock production and increasing concern for animal health and better welfare practices (Oliveira et al., 2021c). In our previous work (Oliveira et al., 2021a), sawgrass aqueous/acetone extracts were rich in total phenolics and tannins, and exhibited a significant activity, especially those made from biomass collected in summer and autumn. Such results, especially the tannins levels, prompt us to evaluate the antiparasitic properties of such extracts collected along the year toward larvae and eggs from two relevant GINs, namely *H. contortus* and *T. colubriformis*. *Haemonchus contortus* reside in the abomasum, while *T. colubriformis* exist in the small intestine, and both reduce voluntary feed intake and nutrient absorption, thus reducing drastically the production of small ruminants (Hoste et al., 2016).

The extracts were more active toward larvae than eggs, most probably due to structural dissimilarities between the eggshell and the larval sheath (Mansfield et al., 1992; Hoste et al., 2015). Differences in susceptibility between eggs and adults are also attributed to the chemical components in the extracts (Araújo-Filho et al., 2018). The extracts were also more active toward *H. contortus*, which is consistent with the higher susceptibility of *H. contortus* in contrast to *T. colubriformis*, reported previously by other authors (Paolini et al., 2004; Brunet and Hoste., 2006; Quijada et al., 2015). Seasonal differences in bioactivity were observed mainly for summer and autumn samples, we questioned whether it could be (1) due to environmental effects, since the production of these metabolites is part of the plant defense machinery, to cope with the harsh settings of the dry Mediterranean climate (Di Ferdinando et al., 2014); or (2) due to differences in biochemical contents associated to the plant organs, viz. leaves and inflorescences. Thus, we proceed to investigate the *in vitro* anthelmintic properties of extracts made from these organs collected in summer (Table 2). The inflorescences extracts were more effective than leaves against egg hatching, for both parasite species (Table 2). Interestingly, despite the high activity of inflorescences sample on EHIA, this is not reflected in the seasonal samples, particularly in summer as marked differences are not observed between samples collected in different seasons. Having in mind that an extract is a complex mixture of compounds, the latter observations can be a result of synergistic/antagonist interactions between the present metabolites or perhaps due to a dilution of the compounds of interest in the mixture (leaves and inflorescences in the summer sample) in comparison to the inflorescences extract alone.

Aiming to elucidate the role of polyphenols in the anthelmintic properties, the extracts were retested after and before treatment with PVPP, a polyphenol-binding agent. All PVPP-treated extracts retained activity toward *H. contortus* and *T. colubriformis* larvae, and egg hatching was completely restored (Figures 2–4), which agrees with previous results obtained with the summer sample (Oliveira et al., 2021b). Thus, polyphenols seem to be the main metabolites involved for the egg hatching inhibitory properties of the extracts at the highest concentration (2500 μg mL^–1^). In contrast, besides polyphenols, other compounds not adsorbed by PVVP seem also effective on L3 larvae exsheathment at 1,200 μg mL^–1^.

An HPLC-ESI-MSn comparative analysis coupled with the use of PVPP was conducted in an attempt to answer the following questions: (1) which major metabolites, removed after PVPP treatment, may be identified to exert the egg hatching inhibitory effects?; (2) which compounds may be remaining after PVPP treatment that can account for the larvae exsheathment activity?; and (3) what chemical variations occur between leaves and inflorescences extracts that may justify the significant higher anthelmintic activity of the latter samples ? Our previous results obtained for the summer samples (Oliveira et al., 2021b) provided some hints to address the first two questions, yet seasonal fluctuations and organ related variations were not priorly considered. We expected that an in-depth comparative analysis of the chemical assets of seasonal and organ extracts, in combination with the biological data herein presented, enables a clarification of the bioactive metabolites of interest for AH properties and its production dynamics. Flavonoids were the dominant compounds identified in all the extracts, especially flavan-3-ols (epigallocatechin, epicatechin **14**), proanthocyanidins (condensed tannins), luteolin, and glycosylated flavonoids (Table 4). Summer and autumn samples had the highest amounts of epicatechin **14** and luteolin **29**. Differences on the polyphenolic composition of plants have been demonstrated to be correlated to environmental changes, particularly in Mediterranean plants subjected to drought, high temperature and solar irradiance, high UV intensity and salt stress conditions, expected during dry seasons such as summer and autumn (Hernández et al., 2004; Di Ferdinando et al., 2014; Gori et al., 2020). This data sustains the role of polyphenols in plant defensive and adaptative strategies to cope with the challenging Mediterranean environmental settings. For example, quercetin and luteolin derivatives act as photoprotector compounds and accumulate in response to UV radiation and increased sun irradiance (Tattini et al., 2004; Agati et al., 2011). Nevertheless, in the wild, besides environmental variations, other abiotic and biotic factors interact concomitantly, which can also affect the production of these metabolites, e.g., the phenological stage of the plant. In this sense, we suspected that organ-related variations may also account for the increased **14** and **29** levels observed for these dry seasons, since the inflorescences extract (from the summer sample) also exhibit increased amounts of **14** and **29**, in contrast to leaves, supporting the former observed variations in the anthelmintic activity. Of interest is the proanthocyanidin dimer + (epi)gallocatechin 3 + 4 content in autumn, but it did not influence greatly the anthelmintic properties. In fact, high amounts of 3 + 4 are noted in all extracts except for inflorescences. Since the inflorescences extract was particularly active against GIN egg hatching, most probably these compounds are not significantly impacting the anthelmintic effects of the extracts. On the other hand, the anthelmintic value of proanthocyanidins is well-recognized in the scientific community (Mueller-Harvey et al., 2018).

Proanthocyanidins **7** and **9–13** were identified but not quantified due to low signal, limiting a complete elucidation of the contribution of tannins to the anthelmintic effects, which were present in all samples, and particularly in the summer and inflorescences extracts. Moreover, other unidentified metabolites, particularly present in inflorescences extract might also contribute to the observed effects. The *in vitro* anthelmintic effects of flavan-3-ols, proanthocyanidins and luteolin are well-described in the literature (Molan et al., 2003; Klongsiriwet et al., 2015; Quijada et al., 2015). Epicatechin and epigallocatechin suppress *T. colubriformis* larvae development by 50% at 43 μg mL^–1^, but were less effective on egg hatching, inhibiting less than 20% up to 1 mg mL^–1^ (Molan et al., 2003). Moreover, Soldera-Silva et al. (2018) reported that epicatechin exhibit an IC_50_ value of 10 μg mL^–1^ in the larval migration test, using *H. contortus* parasites (Soldera-Silva et al., 2018). Regarding proanthocyanidins, several plant extracts rich in these metabolites have documented anthelmintic effects, influenced by the concentration, polymer size, and structural composition of these complex molecules. The most common monomers of proanthocyanidins are catechin and epicatechin [proacyanidin (PC)-type tannins] and gallocatechin and epigallocatechin [prodelphinidin (PD)-type tannins]. Interestingly, it has been demonstrated that PD are more potent inhibitors than PC on L3 exsheathment of *H. contortus* and *T. colubriformis* (Brunet and Hoste, 2006; Quijada et al., 2015; Mueller-Harvey et al., 2018) or egg hatching of *T. colubriformis* (Molan et al., 2003), and that the addition of a galloyl group enhances activity (Molan et al., 2003). In addition to these catechin derivatives, the flavonoid luteolin **29** exhibited an IC_50_ value of 17.1 μg mL^–1^ against L3 larvae exsheathment of *H. contortus* (Klongsiriwet et al., 2015). Synergistic interactions between procyanidins and luteolin (30 μM) have been demonstrated previously, resulting in a reduced IC_50_ value of 75.9 μg mL^–1^, in comparison to the isolated procyanidin fraction (IC_50_ = 356 μg mL^–1^; Klongsiriwet et al., 2015). However, more recently, **29** was ineffective as a larvicidal agent at 2.5 mg mL^–1^, against L3 larvae of *H. contortus* (Delgado-Núñez et al., 2020). These former results emphasize that flavan-3-ols, proanthocyanidins and luteolin are most likely the bioactive metabolites of *C. mariscus* extracts, as they are some of the main metabolites present. However, the contribution of flavonoid glycosides cannot be excluded, particularly because they were detected in high amounts in all extracts, except for inflorescences. Still, the considerably lower amount of flavonoid glycosides in the inflorescences extract perhaps may have unmasked the interactions with other metabolites present (e.g., proanthocyanidins and luteolin) leading to improved anthelmintic effects. Flavonoid glycosides have been identified in the chemical profile of several plant species with proven anthelmintic effects (Barrau et al., 2005; Mengistu et al., 2017; Araújo et al., 2019; Romero et al., 2020), and some authors have posed the possibility of occurring interactions of this type of compounds with GIN (Barrau et al., 2005; Alonso-Díaz et al., 2008). Barrau et al. (2005) determined that 3 flavonol glycosides (quercetin-3-*O*-rutinoside or rutin, kaempferol-3-rutinoside or nicotiflorin and isorhamnetin-3-rutinoside or narcissin) reduced the migration of *H. contortus* L3 larvae in 25–35% when applied at 1,200 μg mL^–1^. Moreover, Sprengel Lima et al. (2021) observed that the addition of rutinose to the quercetin structure resulted in a 2-fold increase in the larvicidal activity. In addition, two flavone-*C*-glycosides (isoschaftoside and schaftoside) exhibited high toxicity against the parasitic nematode Meloidogyne incognita, leading to the death of 50% of the worms at 114.66 and 323.09 μg mL^–1^, respectively (Du et al., 2011). Despite these limited studies, the anthelmintic value of glycosylated forms of polyphenols remains to be elucidated as well as their interactions with aglycone molecules.

The partial inhibition of L3 larvae exsheathment in the PVPP-treated samples could be, in fact, due to the remaining content of flavonoid glycosides identified in the chemical analysis (**15–17, 19–20**). Two C-glycosyl flavonoids were quantified in higher amounts i.e., luteolin-C-Hex-C-Pen 15 and apigenin-C-Hex-C-Pen 16 (Table 5), suggesting its potential involvement in the anthelmintic effects. The quantification of these metabolites in PVPP-treated samples did not vary greatly, except for the inflorescences extract (Table 5). Still, other metabolites present after PVPP treatment remain to be identified (e.g., compound **8**). PVPP is widely used to adsorb polyphenolic structures from plant extracts and consequently to assess its impact on the biological activity (Barrau et al., 2005; Alonso-Díaz et al., 2008; Manolaraki et al., 2010; Vargas-Magaña et al., 2014; Mengistu et al., 2017). However, it is important to keep in mind that PVPP is generally more efficient in binding with molecules with a higher number of hydroxyl groups and that this binding is also influenced by structural substitution patterns (Doner et al., 1993). For example, Laborde et al. (2006) showed that the association of PVPP with quercetin aglycone was 4 to 5-fold stronger than that with its glucoside (quercetin-3-*O*-glucoside).

## Conclusion

Altogether, the results of this work emphasize (1) that many factors can influence the variations in the bioactive compounds explaining the anthelminthic properties of plants; (2) the relevance of considering abiotic factors when surveying botanical resources from the wild; (3) the potential of sawgrass as a source of bioactive metabolites against gastrointestinal nematodes, and (4) a confirmation of the anthelmintic value of a range of polyphenols in the search for alternative control options for GIN infections of ruminants. The current obtained results encouraged future work to focus on the complete elucidation of biological effects of polyphenolic glycosides, but also to explore further the interactions with other phenolic compounds and their contribution for the anthelmintic effects when mixed. In fact, we would suggest that one should be careful when assuming that the remaining activity on anthelmintic assays using PVPP treated samples is not only associated with polyphenolic compounds, and a detailed biochemical characterization by high power analyzing methods before and after the use of PVPP could help to elucidate the bioactive metabolites of interest. In addition, the egg hatching properties of luteolin could be appraised, for a better understanding of the efficient inhibition of egg hatching by the inflorescence extracts.

## Data Availability Statement

The raw data supporting the conclusions of this article will be made available by the authors, without undue reservation.

## Author Contributions

MO performed the design of the study, collection and extraction of plant material, preparation of the extracts, *in vitro* assays, and wrote the first draft of the manuscript. CL assisted in the anthelmintic assays. EL-M performed the HPLC-ESI-MSn analysis. HH performed the design of the study and review of the manuscript. LC performed the design of the study, review and final approval of the manuscript, and was responsible for funding acquisition. All authors contributed to the article and approved the submitted version.

## Conflict of Interest

The authors declare that the research was conducted in the absence of any commercial or financial relationships that could be construed as a potential conflict of interest.

## Publisher’s Note

All claims expressed in this article are solely those of the authors and do not necessarily represent those of their affiliated organizations, or those of the publisher, the editors and the reviewers. Any product that may be evaluated in this article, or claim that may be made by its manufacturer, is not guaranteed or endorsed by the publisher.
